# Classification of primary progressive aphasia: challenges and complexities

**DOI:** 10.12688/f1000research.21184.1

**Published:** 2020-01-30

**Authors:** Donna C. Tippett

**Affiliations:** 1 Departments of Neurology, Otolaryngology - Head and Neck Surgery, and Department of Physical Medicine and Rehabilitation, Johns Hopkins University School of Medicine, Baltimore, Maryland, 21287, USA

**Keywords:** primary progressive aphasia, logopenic variant primary progressive aphasia, nonfluent agrammatic primary progressive aphasia, semantic varaint primary progressive aphasia

## Abstract

Primary progressive aphasia (PPA) is classified into three variants, logopenic variant PPA (lvPPA), nonfluent agrammatic PPA (nfaPPA), and semantic variant PPA (svPPA), based on clinical (syndromic) characteristics with support from neuroimaging and/or underlying neuropathology. Classification of PPA variants provides information valuable to disease management. International consensus criteria are widely employed to identify PPA subtypes; however, classification is complex, and some individuals do not fit neatly into the subtyping scheme. In this review, diagnostic challenges and their implications are discussed, possible explanations for these challenges are explored, and approaches to address PPA classification are considered.

## Introduction

Primary progressive aphasia (PPA) is a neurodegenerative clinical syndrome characterized by the predominance and insidious onset of language impairments, and gradual deterioration of these abilities over time, associated with atrophy of the language network of the brain, including frontal, temporal, and parietal regions of the left hemisphere ^1– 3^. PPA is classified as a rare disease (defined as a condition which affects fewer than 200,000 people in the United States) by the Genetic and Rare Diseases Information Center of the National Center for Advancing Translational Sciences (NCATS) ^4^. Although it is an uncommon diagnosis, PPA has considerable personal and societal impact, as it affects individuals in the prime of their lives, compromising their ability to work and engage in home and community life. Three different PPA variants are specified by international consensus criteria based on clinical presentation (language manifestations) with support from patterns of atrophy and/or underlying neuropathology: logopenic variant PPA (lvPPA), nonfluent agrammatic PPA (nfaPPA), and semantic variant PPA (svPPA) ^3^. Individuals with lvPPA are distinguished from those with other variants by impaired single-word retrieval in spontaneous speech and naming, impaired repetition of phrases and sentences ^5– 7^, and left temporo-parietal atrophy ^5, 8, 9^. Those with nfaPPA demonstrate nonfluent, effortful speech and agrammatism ^5, 10– 13^; atrophy is characteristically present in the inferior frontal gyrus (IFG) and prefrontal/premotor regions and to a lesser extent in the posterior temporal regions in this variant ^5, 14– 17^. Those with svPPA, a disorder of semantic memory, present with impaired object knowledge, anomia, single-word comprehension deficits ^5, 18, 19^, and atrophy in the anterior parts of the temporal lobe (ATL) ^5, 9, 20, 21^. The variants svPPA and nfaPPA are typically associated with pathologies in the spectrum of frontotemporal lobar degeneration (FTLD) ^9, 22– 29^; lvPPA is most frequently associated with Alzheimer pathology ^7, 30– 32^.

Although there are no interventions currently available to cure PPA, research is underway to optimize PPA diagnosis and management. At present, intervention is primarily speech-language pathology treatment, behavioral management, and education/counseling of patients, family members, and caregivers ^33^. Appropriate management depends on the accurate diagnosis of PPA variants, as each of the variants presents with different trajectories and manifestations. Knowing the projected clinical course, based on differential diagnosis of PPA variant, enables healthcare providers to make recommendations about future needs. In this review, diagnostic challenges and their implications are discussed, possible explanations for these challenges are explored, and approaches to address PPA classification are considered.

## Classification can be challenging

International consensus criteria include inclusion and exclusion criteria to establish the diagnosis of PPA and expressive and receptive language characteristics, atrophy patterns, and underlying neuropathologies to diagnose specific variants ^3^. These criteria facilitate communication among clinicians and researchers and have been widely adopted for classifying PPA variants, as shown by more than 2,000 citations referencing the international consensus criteria in published manuscripts (see here). The expert author panel intended for the classification system to be used in the early stages of disease progression because distinctions between PPA variants can become obscured with the variants becoming more alike over time ^34, 35^. Tasks, such as word–picture matching to test single-word comprehension or oral picture description to investigate grammar, are identified to discern PPA variants in the clinical setting ^3, 36, 37^. Henry and Grasso ^38^ and Marshall and colleagues ^39^ provide explicit guidance for clinicians to assess speech, language, and cognition in individuals with PPA, including predicted performance on tests by PPA variant.

Nevertheless, differential diagnosis of PPA can be challenging. Since publication of the international consensus criteria, researchers have applied these guidelines to diagnose individuals with PPA with varying degrees of success. Harris *et al*. ^40^ reported that five out of 30 (17%) individuals, with early onset of disease, who met the criteria for PPA did not meet the criteria for any of the variants. Wickland *et al*. ^41^ reported that 26 out of 84 individuals with PPA (31%) (mean disease duration = 2.5 years, range 2.0–3.9 years) did not meet minimum diagnostic criteria for classification of any variant and consequently were labeled “unclassified”. Sajjadi, Patterson, Arnold, Watson, and Nestor ^42^ applied the international consensus criteria to 46 individuals with PPA (disease duration = 2–7 years) and found that 19 of their participants (41%) did not satisfy the diagnostic criteria for any of the three proposed variants and thus represented mixed PPA. These findings suggest that while the variant of most individuals with PPA can be classified according to the Gorno-Tempini *et al*. ^3^ paradigm, the presentation of some individuals may not fit neatly into any one of the three clinical syndromes and may represent either individuals who cannot be grouped together as representing another variant of PPA or individuals who have disparate language disorders.

## Why is classification so hard?

The identification of PPA variants can be complicated for a variety of reasons; however, three contributors are the seeming overlap of language characteristics among variants, speech and language features which can obscure differential diagnosis, and variability in clinical presentation, especially in lvPPA and nfaPPA. One example of apparent commonality in language deficits is impaired naming, which is present in all three PPA variants; another example is impaired repetition, which is seen in both lvPPA and nfaPPA. The underlying causation of these language manifestations is different, and recognition of the underlying mechanism of overt speech and language errors is vital to diagnosis. With respect to naming impairment or anomia, individuals with lvPPA may have impaired access to the representations of words in the spoken modality, those with svPPA have impaired access to modality-independent semantics (word meaning), and those with nfaPPA may have either impaired access to the spoken-word form or impaired motor speech ^43^. Likewise, the source of impaired repetition is variant specific: impaired working memory contributes to impaired repetition of phrases and sentences in lvPPA, whereas apraxia of speech compromises repetition in nfaPPA. Apraxia of speech can be demonstrated on single-word-level repetition tasks in nfaPPA, although in the early stages of disease, errors may emerge only during repetition of lengthy or complex words, phrases, and sentences.

Furthermore, differential diagnosis can be problematic for diagnosticians. Agrammatism, a core component of nfaPPA, may be difficult to detect in the presence of concomitant apraxia of speech. Also, the distinction between phonetic (apraxia of speech) and phonemic speech sound errors is challenging for even experienced clinicians.

In addition, the PPA variants differ in the consistency of their clinical presentation. Those with lvPPA and nfaPPA have more variability in contrast to those with svPPA ^41, 42^. Individuals with svPPA present with the hallmark characteristics of impairments in single-word comprehension (e.g. difficulty selecting a pictured item from an array when told the object label) and semantic knowledge (e.g. difficulty demonstrating the use of a common object or difficulty identifying pictured objects which “go together”, such as “pencil and paper” versus “pencil and car”). Hoffman *et al*. ^44^ demonstrated that svPPA is a clearly defined subtype of PPA whereas the lvPPA and nfaPPA profiles are less distinct. They identified three PPA clusters: one which closely corresponds to svPPA with bilateral anterior temporal lobe (ATL) atrophy (left greater than right), another which includes features of both lvPPA and nfaPPA, and a mixed PPA group characterized by weak semantic abilities and severe impairments in speech production, repetition, and syntax (not attributable to more advanced disease). In the non-svPPA groups, patterns of atrophy were widely distributed.

Variability in nfaPPA is also manifested in that a subset of those with this variant exhibit single-word comprehension deficits in addition to apraxia of speech and/or agrammatism, representing a fourth or mixed variant of PPA not recognized in current international consensus guidelines for diagnostic classification ^13, 45, 46^. Schaeverbeke *et al*. ^47^ found that seven out of 12 individuals with *a priori* diagnosis of nfaPPA demonstrated single-word comprehension deficits, consistent with a mixed variant PPA, and found that those with this mixed presentation had deficits on object knowledge and object recognition relative to healthy controls, but to a lesser degree than those with svPPA.

Variability in both clinical presentation and neuroimaging is described in association with lvPPA. Sajjadi, Patterson, and Nestor ^48^ found a pattern of left temporoparietal atrophy, most similar to that seen in lvPPA, in 14 individuals with mixed PPA. They concluded that Alzheimer’s pathology, the underlying etiology of lvPPA, can result in a heterogeneous language profile in a PPA subtype that is neither nfaPPA nor svPPA. The concept of a logopenic spectrum has been advanced, which includes lvPPA as defined by consensus guidelines and lvPPA+ and lvPPA– defined as clinical phenotypes that are partially consistent with consensus guidelines ^32^. Preiß *et al*. ^49^ found diffuse cortical thickness reductions in the left hemisphere language network in Alzheimer’s disease (AD)-related PPA, including regions characteristically associated with nfaPPA and svPPA. The authors proposed that this finding explains why the language deficit in AD-PPA is often more extensive than is captured by the consensus guidelines for diagnosing lvPPA. Furthermore, disease duration complicates diagnosis. The clinical profiles of the PPA variants do not remain mutually exclusive as the disease process advances.

## Why is classification important?

Although classification of PPA variant by fluid biomarkers is promising for establishing underlying pathology in the future ^50^, at present, classification which relies on clinical characteristics and neuroimaging is the gold standard for care. Classification is not of purely theoretical interest; it has important practical implications for developing treatment recommendations and planning for future anticipated needs. Individuals with PPA and their caregivers want, and need, prognostications regarding functional milestones: time to loss of occupational, social, and physical independence. Longitudinal investigations of the evolving course of PPA, rather than cross-sectional studies, can help to address patients’ and families’ questions in the absence of a reliable clinical staging system for PPA at present. Longitudinal decline in PPA has been well documented, enabling healthcare providers to counsel individuals with PPA and their families and caregivers about decline specific to each variant ^34, 35, 51– 55^. Sebastian *et al*. ^56^ investigated longitudinal patterns of decline in naming and semantic knowledge in PPA variants and examined the effects of other variables on the rate of decline. They found that nfaPPA had the most precipitous rates of decline in oral naming, followed by svPPA, then lvPPA, in individuals with similar disease duration at baseline testing. This decline was in part due to apraxia of speech in nfaPPA. Female sex, longer symptom duration, higher baseline test score, and speech-language rehabilitation were associated with slower decline.

PPA variant also informs the nature of treatment to be provided. Speech-language pathology intervention is the mainstay treatment for PPA ^57– 61^, although cholinesterase inhibitors and/or memantine may be used in lvPPA because of its underlying AD pathology. Differential response to treatment may be seen in PPA. For example, transcranial direct cortical stimulation (tDCS) combined with written-language therapy was beneficial in those with lvPPA and nfaPPA; tDCS did not confer an advantage for those with svPPA ^62^.

## Can the diagnostic process be refined?

Although the current consensus criteria capture the majority of individuals with PPA, a minority of individuals with PPA cannot be classified by these guidelines. Refinements of the diagnostic process may include acknowledgement of mixed, atypical, or stratified designations and incorporation of other language and nonlanguage characteristics of PPA in the classification structure. Schaeverbeke *et al*. ^47^ recommended that less-restrictive criteria regarding single-word comprehension and object knowledge for nfaPPA should be considered rather than adding a fourth PPA variant because the neurobiology underlying nfaPPA and mixed PPA was similar (i.e. elevated [18F]-THK5351 binding in the supplementary motor area and left dorsal premotor cortex).

Inclusion of the reading and writing profiles in all PPA variants may enhance classification. The Gorno-Tempini *et al*. ^3^ consensus criteria include surface dysgraphia (i.e. difficulty spelling irregular words, such as “niche” or “yacht”) as a diagnostic criterion for svPPA. It is known that those with nfaPPA have greater difficulty spelling pseudowords (e.g. stiable and janiliation) than real words and greater difficulty spelling irregular than regular words and those with lvPPA present with features of both svPPA and nfaPPA ^63^. Utianski *et al*. ^64^ found that their cohort of unclassifiable PPA patients had more difficulty reading and writing nonwords than irregular words and more difficulty writing than reading both nonwords and irregular words, noting that reading and writing test performance was the only abnormal finding for some individuals. These authors endorsed reading and writing measures in future diagnostic paradigms. Neophytou *et al*. ^65^ capitalized on the spelling patterns of words versus pseudowords in the three PPA variants and employed sophisticated statistical analysis and automated classification to distinguish PPA variants. Classification accuracy was 70% for nfaPPA but only 66% for svPPA and 59% for lvPPA using their approach, which they described as a “proof of concept” rather than a clinical tool at this juncture. Classification of lvPPA remained most challenging.

Investigation of nonlanguage cognitive abilities and behaviors in lvPPA may be helpful in this regard. For example, those with lvPPA demonstrate difficulties in verbal working memory ^5^, which can be assessed using a digit span task. Visuospatial abilities and visual memory also have been studied in PPA, revealing deficits in this patient population, especially in lvPPA ^66– 69^. Tippett *et al*. ^70^ found that delayed figure copying was relatively spared in nfaPPA and significantly more impaired in lvPPA and svPPA. They also found an association between PPA variants and scores on immediate figure copying with a greater percentage of those with nfaPPA scoring within normal limits than those with lvPPA and svPPA. There is increased awareness of nonverbal auditory and other extra-linguistic features in PPA, especially in nfaPPA and svPPA. Individuals with nfvPPA demonstrate prominent deficits of early perceptual auditory analysis including impaired temporal (rhythm) perception, and individuals with svPPA exhibit auditory associative deficits and impaired sound meaning, consistent with their respective speech and language characteristics in the Gorno-Tempini classification paradigm ^71, 72^. In a related, more recent study, individuals with nfaPPA were found to have poorer performance on pure-tone audiometry than healthy older individuals or individuals with AD ^73^. Comprehensive audiologic evaluation, while likely not a routine consultation at present, may be a valuable addition to the medical work up of individuals with PPA. Beyond the clinical setting, further investigation of auditory processing deficits in PPA may reveal new insights regarding nonverbal manifestations of PPA and novel approaches to, and perhaps revision of, the current conceptualization of PPA as a language-led dementia ^74, 75^.

It is important to note that statistically significant differences in performance between PPA variants on various measures in a research context do not necessarily translate to diagnostic utility in a clinical context. These differences need to be explored further to determine sensitivity, specificity, and positive and negative predictive values of proposed diagnostic measures. In addition, one must ensure that the differences on tasks reported as distinguishing the PPA variants are not also the tasks used to diagnose the PPA variants. For example, if one wishes to investigate single-word comprehension in the PPA variants by examining performance on a spoken-word recognition task, then another task, such as a word–picture verification measure, must be used to diagnose spared versus impaired single-word comprehension. An alternative approach is to investigate language profiles in individuals grouped by neuropathology. Xiong *et al*. ^76^ found that anomia tended to be manifested by “don’t know” responses by individuals in their AD group and by word substitutions in their FTLD-spectrum group. The authors caution that these findings are preliminary and require further study to determine if language profiles can predict underlying pathology.

Behavioral manifestations are another means to distinguish the PPA variants. On the Frontal Behavioral Inventory (FBI) ^77– 80^, restlessness appears to be more characteristic of svPPA; more severe personal neglect is seen in the svPPA and nfaPPA groups than in the lvPPA group, and more severe impairment in judgment distinguishes the nfaPPA group from the lvPPA group ^81^. In addition, language performance and behavioral disturbances are correlated in lvPPA but not other PPA variants. Negative behaviors do not develop in lvPPA until language deficits are severe ^82^. Use of a caregiver questionnaire, such as the FBI or the modified version of this inventory ^83^, may yield useful information to aid PPA diagnosis in the clinical setting.

Classification relies on expert, comprehensive evaluation. Documentation of an overt behavior (e.g. impaired naming or impaired repetition) is likely not sufficient for differential diagnosis; rather an understanding of the underpinnings of the manifestations of impaired performance on a speech and language task is necessary to ascertain a correct diagnosis. Marshall and colleagues ^39^ provide a roadmap for PPA diagnosis in the clinical setting along with recommendations for tasks which aid in uncovering the mechanism for seemingly similar impairments. For example, clinicians are advised to assess diadochokinesis (rapid repetitions of syllable sequences, such as “puh-tuh-kuh”) and digit recall to reveal apraxia of speech in nfaPPA and impaired working memory in lvPPA, which can account for repetition impairment.

The use of sensitive and specific tests is needed for the assessment of PPA. There is likely heterogeneity in tests and cutoffs used in the diagnosis of PPA across different clinical and research sites. Normative data from the National Alzheimer’s Coordinating Center website (see here) can discriminate test performance on many measures between individuals with PPA and healthy controls ^84^. Several recently published assessment batteries are specifically designed for the assessment of PPA, such as the Progressive Aphasia Severity Scale ^85^ and the Sydney Language Battery ^86^. The Progressive Aphasia Severity Scale is a clinician rating scale of speech, language, and functional communication based on the results of a structured interview with patients and caregivers and a caregiver questionnaire. The Sydney Language Battery is a brief battery of tasks (picture naming, word comprehension, semantic association, and repetition) designed to differentiate among PPA subtypes. Timing of test administration and severity of disease may need to be considered as well. With disease progression, the ability to perform valid assessments of individuals with PPA and determine variant may diminish over time ^34^. Much like stroke aphasia classification, which includes the designation of global aphasia to describe impairment in all language modalities, the designation of global PPA in those with end-stage disease may be a diagnostic entity worth considering.

## Conclusion

The classification of PPA variants is complex, and several factors contribute to this challenge. While the importance of accurate diagnosis is indisputable, approaches to improve differential diagnosis—for example, recognition of a fourth variant of PPA, stratified designations, and modification of diagnostic criteria—remain controversial. Valid, comprehensive assessment of individuals with PPA remains central to the characterization of speech, language, and behavioral manifestations of PPA. Collaboration in clinical and research arenas is needed to address the theoretical and practical aspects of PPA nosology.
